# The Curious Case of a Pilonidal Sinus of the Sole of the Foot Mimicking a Painful Corn: An Unusual Entity

**DOI:** 10.1055/s-0044-1801785

**Published:** 2025-01-13

**Authors:** Alexander George

**Affiliations:** 1 Department of Plastic Surgery, Ernakulam Medical Centre, Cochin, Kerala, India

The term “pilonidal sinus,” first described by Herbert Mayo in 1833, and later named “pilonidal sinus” by Hodges in 1880, brings to our mind the ever-suffering patient with a discharging, painful, and nonhealing lesion in the sacrococcygeal area. 1 However, a number of other sites being affected have also been reported in the past: axilla, groin, interdigital web, umbilicus, nose, clitoris, prepuce, penis, occiput, intermammary areas, suprapubic area, and feet. 2 In the foot, although interdigital pilonidal sinus of the toes are common, pilonidal sinus of the sole of the foot is a rare occurrence and only a few cases have been reported in the literature to date.

**Fig. 1:**
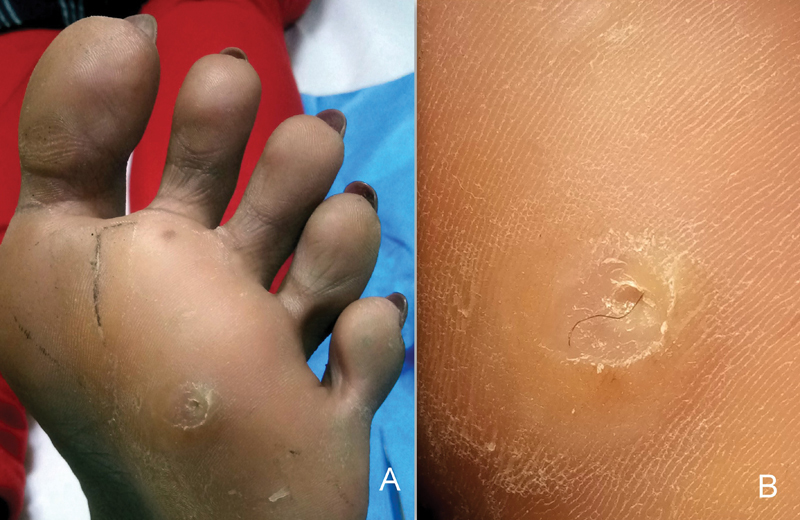
( **A** ) The sole lesion giving the appearance of a plantar corn. ( **B** ) A pilonidal sinus with the hair embedded in it.

A 32-year-old lady came to our department with history of a painful lesion over the sole of her foot. For over a month, the lesion had been troubling her and she was being treated elsewhere as a corn on the foot with corn cap applications. In order to confirm the diagnosis, I decided to pare the lesion and to my surprise found a hair almost a centimeter and half extending into the soft tissue surrounded by infected tissue ( Fig. 1 ). An offer of surgical excision of the lesion was placed before the patient, which was unfortunately refused as she wished to get the hair and infected elements cleared along with antibiotics administration. The acute inflammatory reaction settled down over a few days and the patient seemed temporarily satisfied and defiant, in the face of a possible recurrence.

An inquiry into her profession revealed that she was a hairdresser. The custom of removing shoes in a lot of shops and workplaces in Kerala and walking barefoot could have probably promoted this “hair” attack to her sole. Barbers, hairdressers, dog groomers, sheep shearers, and those who constantly come in contact with hair and wool are prone to develop a pilonidal sinus. It is believed that the freshly cut short hairs pierce the sole of the foot and serve as a focus for inflammation, foreign body reaction, and infection, thereby resulting in abscess, sinuses, and cysts. 3 The rare possibility of a cancerous transformation in a long standing pilonidal sinus is also a cause of concern. 4 The sole of the foot is an unusual area for a pilonidal sinus and we need to create awareness in patients, doctors, and medical textbooks about this unusual site to avoid misdiagnosis and offer appropriate treatment.
